# Internet of Things and Enhanced Living Environments: Measuring and Mapping Air Quality Using Cyber-physical Systems and Mobile Computing Technologies

**DOI:** 10.3390/s20030720

**Published:** 2020-01-28

**Authors:** Gonçalo Marques, Nuno Miranda, Akash Kumar Bhoi, Begonya Garcia-Zapirain, Sofiane Hamrioui, Isabel de la Torre Díez

**Affiliations:** 1 Polytechnic Institute of Guarda, 6300-559 Guarda, Portugal; nuno-miranda@sapo.pt; 2 Institute of Telecommunications, University of Beira Interior, 6200-001 Covilhã, Portugal; 3 Department of Electrical & Electronics Engineering Sikkim Manipal Institute of Technology (SMIT), Sikkim Manipal University (SMU), Sikkim, 737136 Majhitar, India; akash.b@smit.smu.edu.in; 4 eVIDA research group, University of Deusto, Avda/Universidades 24, 48007 Bilbao, Spain; mbgarciazapi@deusto.es; 5 Polytech School, University of Nantes, CNRS, IETR UMRS 6164, 85000 La Roche-sur-Yon, France; sofiane.hamrioui@univ-nantes.fr; 6 Department of Signal Theory and Communications, and Telematics Engineering University of Valladolid 12 Paseo de Belén, 15, 47011 Valladolid, Spain; isator@tel.uva.es

**Keywords:** air quality, enhanced living environments, internet of things, mobile computing, mobile health

## Abstract

This paper presents a real-time air quality monitoring system based on Internet of Things. Air quality is particularly relevant for enhanced living environments and well-being. The Environmental Protection Agency and the World Health Organization have acknowledged the material impact of air quality on public health and defined standards and policies to regulate and improve air quality. However, there is a significant need for cost-effective methods to monitor and control air quality which provide modularity, scalability, portability, easy installation and configuration features, and mobile computing technologies integration. The proposed method allows the measuring and mapping of air quality levels considering the spatial-temporal information. This system incorporates a cyber-physical system for data collection and mobile computing software for data consulting. Moreover, this method provides a cost-effective and efficient solution for air quality supervision and can be installed in vehicles to monitor air quality while travelling. The results obtained confirm the implementation of the system and present a relevant contribution to enhanced living environments in smart cities. This supervision solution provides real-time identification of unhealthy behaviours and supports the planning of possible interventions to increase air quality.

## 1. Introduction

Internet of Things (IoT) paradigm is related to the connection of physical objects to the Internet. These objects should be pervasive and ubiquitous by supporting reliable sensing capabilities. Moreover, these devices can be handled via unique addresses, support cooperation capabilities and provide ubiquitous and pervasive applications [1,2]. There is clear evidence of the increase of IoT systems and their adoption for several daily routine activities. People’s homes are being invaded by IoT products for surveillance, environmental monitoring, energy consumption analysis and home automation. Regarding the healthcare field, IoT technologies provide effective and efficient methods for enhanced living environments (ELE) and wellbeing [3]. The IoT systems will provide a relevant evolution of the healthcare field and will bring several social benefits [4,5]. Numerous activities that, in the past, could only be done by healthcare staff can be automated using IoT technologies. Therefore, the cost of healthcare can be decreased, and at the same time, IoT systems allow remote patient monitoring and data consulting anytime and anywhere [6]. Furthermore, IoT is closely related to several research fields in this context, such as mobile health (mHealth) and ambient assisted living (AAL).

AAL research programs aim to develop services and products to create ELE to improve health and wellbeing, particularly for the elderly [7,8]. In total, 20% of the population will be aged 60 years or older in 2050 [9]. This will lead to an increase in healthcare service costs, a lack of caregivers and a relevant social impact. AAL projects aim to keep people’s autonomy and independence. Moreover, 87% of people desire to stay in their residences and assume the high cost of nursing care instead of being sent to nursing homes [10].

mHealth is a research field which aims to develop cost-effective healthcare services using mobile computing technologies [11]. The mHealth projects are enhanced healthcare systems which aim to promote the relationship between patient and medical staff [12]. The design of mHealth systems to promote health and well-being has increased in the past few years [13]. The mHealth research programs have to focus on pervasive methodologies to address the user requirements and promote the acceptance of these systems [14].

Combining IoT, AAL and mHealth domains is possible to develop cyber-physical systems (CPS) with significant capabilities for sensing and connecting [15,16,17]. The recent enhancements in embedded systems, networks, sensors and actuators meet the requirements for the development of real-time supervision solutions for ELE [18]. A cyber-physical system is an integration of computing technologies which connect the cyber world to physical processes through communication technologies and is closely related to the IoT concept [19]. CPS are built on top of embedded technologies and incorporate several sensors to monitor and control the physical environment. Moreover, the data collected are stored on remote servers to be processed and analyzed. The CPS allow the creation of remote real-time monitoring solutions for ELE by providing efficient methods for data collection and transmission. However, the development of smart systems for ELE based on IoT and mHealth through CPS has design and implementation challenges such as human–computer interaction, information architecture, interoperability and accessibility [20]. Furthermore, there are also security and privacy issues as the collected data are exceptionally sensible and the confidentiality of that information must be ensured. Despite all the advantages of these systems, technology should never replace human care as human contact is also essential. Technology should be only used as an essential and useful complement to personal care.

Mobile computing technologies in general and smartphones in particular incorporate significant communication technologies as well as a high processing power. On the one hand, smartphones incorporate several sensors for ambient data collection, such as an accelerometer, Global Positioning System (GPS), gyroscope, camera, microphone and a proximity sensor. Moreover, mobile devices support relevant communication protocols such as Near Field Communication (NFC) and Bluetooth Low Energy (BLE). Smartphones are used for several applications related to AAL and mHealth, such as activity recognition and physical activities analysis [21,22]. Smartphones incorporate both short-range such as Bluetooth and Wi-Fi as well as long-range such as GPRS, 3G/4G communication technologies [23]. On the other hand, smartphone usage in the western world has increased. These mobile computing technologies support daily routine tasks and offer a diversity of applications such as communication, education, data visualization and analytics [24]. On the other hand, people typically spend more time using smartphones when compared with other devices, such as personal laptops [25]. Smartphones support a pervasive connection to the Internet, and these devices introduced several changes in people’s daily routine [26].

First, due to socio-economic development, air pollution is increasing in developing countries, which leads to significant health impacts [27,28,29]. The emission of several air pollutants in Europe and the United States has decreased in the past decades [30,31]. Nevertheless, most people live in cities where air quality problems persist since ozone (O_3_), nitrogen dioxide (NO_2_), and particulate matter (PM) exposure values lead to relevant health risks and are estimated to reduce life expectancy [32]. Consequently, air pollution remains a critical problem worldwide [33].

Several epidemiologic research studies state numerous adverse health effects associated with air quality, such as premature death, respiratory, and cardiovascular disease [34]. Air quality assumes a significant responsibility in human exposure to pollutants and is particularly relevant for specific groups such as older adults, students, and people with disabilities [35]. Secondly, numerous research studies noted the negative impacts on health and well-being, particularly on children and older adults related to reduced air quality levels. Poor air quality levels is a critical global health challenge and can be compared to the use of tobacco and sexually transmitted diseases [28]. The Environmental Protection Agency (EPA) stated that indoor air quality (IAQ) pollutants levels can be up to 100 times greater when compared with outdoor air quality and ranked poor air quality in the top 5 environmental risks to global health and well-being [36]. Every year, air quality concentration levels are responsible for 3.2 million deaths and a relevant increase in heart and asthma attacks, dementia, as well as cancer [37,38]. The consequences of poor air quality are most severe in developing countries where there is no regulation to control pollutants emissions. However, air quality levels are also a problem in developed countries. Every year in the USA, approximately 60,000 premature deaths are reported and linked to reduced air quality levels and the healthcare costs related to air quality diseases in healthcare costs reach $150 billion [39]. According to the European Environment Agency, in 2016, air pollution was responsible for 400,000 premature deaths in the European Union (EU). PM caused 412,000 premature deaths in 41 European countries, and 374,000 occurred in the EU [40]. Moreover, the cost related to the air pollutant emissions effect caused by industrial facilities in 2012 has been estimated as at least 59 to 189 billion euros in the EU [41]. Even in locations with good air quality, levels are reported in situations of short-term exposure which conduct relevant health symptoms related to sensitive groups such as elderly and children with asthma and cardiovascular problems [42,43]. Living environments include numerous types of spaces and locations, such as workplaces, clinics, public service centres, faculties, leisure spaces, vehicles, cabins, and outdoor locations [44]. Notably, a significant percentage of indoor environments have a high number of occupants. Taking into consideration all the problems and consequences from reduced air quality exposure, the indoor and outdoor air should be monitored in real time using CPS to improve the health and well-being of the occupants [45]. Furthermore, specific legislation requirements must be created to act in real time in order to promote public health and well-being. By applying efficient and effective monitoring methods, it is possible to identify inadequate air quality concentrations in useful time and plan interventions for ELE and well-being [46]. Regarding the afore-mentioned facts, air quality sensing is a relevant public health problem which must be addressed by several fields of research but mainly by the technological field. Air quality sensing is a relevant factor to be monitored and controlled not only inside buildings but also inside vehicles. Scalable, modular and easy-to-install methods are required to detect poor air quality scenarios.

Carbon dioxide (CO_2_) is one of the greenhouse gases assumed as the primary cause of global warming and environmental degradation [47]. Moreover, the CO_2_ concentration level is a relevant indicator of air quality conditions [48,49]. It is produced in large quantities, is relatively easy to quantify, and can be used to evaluate the degradation of the air quality as a whole [50,51,52]. Numerous authorities define the thresholds for CO_2_ in different countries [53,54]. Concentrations between 250 and 350 ppm are defined as normal outdoor air levels. The concentration commonly measured for occupied indoor spaces ranges from 350 to 1000 ppm. Poor air quality values for CO_2_ associated with complaints of drowsiness are defined from 1000 to 2000 ppm. From 2000 to 5000 ppm, CO_2_ levels are related to several health symptoms such as headaches and increased heart rate. Values higher than 5000 ppm are associated with unusual air conditions with high levels of other air pollutants.

The primary source of CO_2_ emissions in urban areas is related to the use of fossil fuels such as petroleum, hydrocarbons and natural gas [55,56]. Electricity production is also responsible for the emission of high quantities of CO_2_ if the power source is fossil fuels [57,58]. Moreover, transportation and industry must also be taken into consideration regarding CO_2_ emissions [59].

This document proposes an air quality supervision system based on IoT for ELE. The proposed method incorporates a cyber-physical system for air quality monitoring and applies mobile computing technologies to measure and map the air quality levels taking into account the spatial-temporal information. The proposed architecture incorporates a data acquisition prototype and provides a mobile application for data consulting. This cyber-physical system incorporates an ESP32 microcontroller as the processing and communication unit and uses an MH-Z14 CO_2_ sensor. The collected data are transmitted to a smartphone through BLE, which collects the GPS data and uploads all the information to a backend application. The collected data can also be accessed using the mobile application developed by the authors. Furthermore, this information can be consulted by the building and city manager in real time for air quality assessment. These data can be analyzed to plan interventions to create ELE. The proposed solution is portable and can be easily installed and configured, not only to provide air quality supervision in indoor environments where GPS is available, such as vehicle cabins, but also to monitoring outdoor air quality. Moreover, the proposed solution can be installed on top of vehicles to map and measure the air quality in real time. Numerous air quality monitoring systems have been proposed by several research studies available in the literature. These methods incorporate open-source technologies for processing and data transmission and microsensors for data acquisition, but also provide mobile applications for data consulting [60,61,62,63,64,65,66,67]. However, these methods do not provide a portable solution to monitor air quality inside vehicles and do not correlate the air quality levels with geographic coordinates. The main contribution of this paper is to propose a low-cost IoT method to monitor air quality which can be easily installed and configured to supervise vehicles on the move and store the collected values in a structured database, for supporting decision making on possible interventions to promote public health. The main objectives of the manuscript were to present the design and development of a portable real-time data acquisition system for air quality measurement and mapping and to test the proposed IoT approach and system architecture. Furthermore, another relevant objective was to create a reliable stream of air quality data to assist the decision making on possible interventions for ELE and to support the clinical evaluation by correlating the patient health conditions with the air quality data of their living environment.

The rest of this document is organized as follows: Section 2 introduces the related work; Section 3 describes the methods and materials used in the design and development of the proposed architecture; Section 4 presents the results of the research conducted, and Section 5 concludes the paper.

## 2. Background

Smart cities incorporate technology to increase efficiency, sustainability, and economic development for ELE [68]. The creation of a smart city is based on relevant concepts, such as IoT, CPS and mobile computing technologies to improve health and well-being. Regarding the relevant open issues of present-day urban environments, the smart city concept is an effective method to address these challenges [69]. Moreover, the smart city should be considered as an emerging approach to address relevant urban problems originating from population growth and economic activities [70]. However, the implementation of smart cities has numerous challenges, such as the interoperability of different technologies, but also privacy and ethical problems. IoT architectures can be incorporated in smart cities through the development of new daily routine services to increase city efficiency and sustainability [71]. Furthermore, IoT systems can provide interoperability to smart cities as IoT can be used to develop unified urban scale systems [72].

Smart homes are ELE which lead to several benefits for human life. The main goal of smart homes is to allow and maximize the benefits of technology for everyone in order to improve public health and safety [73]. Recent technology advances allow a decrease in the cost of smart homes and the development of smart environments and intelligent sensors for real-time data acquisition, transmission, and storage [74]. IoT architectures are developed to incorporate smart devices for data collection and mobile computing technologies which are used for data consulting [75]. Smart homes must include air quality sensing for ELE and to promote health and well-being. The smart home design must take into consideration older adults to promote effective care and improve safety [76].

The implementation and correlation of different areas of knowledge, such as smart cities, smart homes, mobile computing, IoT and CPS, will lead to smart living, which will bring multiple benefits for everyone [77]. Smart homes, combined with IoT and mobile computing, already integrate numerous benefits in the smart city context [78]. The real-time sensing features enabled by ELE provide immediate alerts based on environmental conditions sensing, which can automatically trigger appropriate interventions to improve public health and safety. Air quality monitoring systems can be used to provide real-time data in both outdoor and indoor living environments to design intervention strategies in a useful time to increase productivity and well-being. Moreover, these data can be consulted by doctors and city managers to correlate environmental conditions with people’s symptoms for enhanced public health and safety.

Numerous open-source IoT methodologies for air-quality sensing incorporate cost-effective sensors for data collection and mobile applications for data access anywhere and anytime, are proposed by research studies available in the literature. A real-time CO_2_ monitoring system composed of a cyber-physical system for ambient data acquisition and web and mobile software compatibility for data consulting is proposed by [64]. This system is a modular, scalable, and low-cost monitoring system which can be wirelessly connected to the Internet using Wi-Fi. The principal purpose of the proposed method is to provide an effective IAQ assessment to anticipate technical interventions for enhanced health and well-being. The iAir is an IoT system for real-time IAQ supervision based on the ESP8266 microcontroller, which is used as a communication and processing unit [63]. This system incorporates a MICS-6814 metal oxide semiconductor sensor which provides carbon monoxide (CO), ethanol, NO_2_, propane and methane supervision. The proposed method incorporates a mobile application for data access and real-time notifications and is based on open-source technologies. The acquisition system is connected to the Internet through Wi-Fi and provides easy installation features. A smartwatch-based application for IAQ data consulting is proposed by [79]. This solution incorporates a hardware prototype for data collection and transmission and is based on open-source technologies. The processing unit is based on the Arduino UNO microcontroller and several sensors are used for temperature, humidity, CO_2_, light, and PM monitoring. The data collected are stored in the ThingSpeak cloud platform. The smartwatch application provides pervasive and ubiquitous access to real-time notifications. The AirPlus is a real-time indoor environmental quality monitoring system which incorporates mobile computing software for data consulting and notifications [80]. The system sensor unit is composed of a PMS5003ST sensor that can monitor formaldehyde (CH_2_O), temperature, relative humidity and PM. The proposed architecture incorporates an acquisition system for data sensing purposes and wireless communication and smartphone application for data visualization and analytics. The main goal is to provide an efficient dataset to plan interventions to improve residents’ productivity and health. Moreover, this dataset can be accessed to associate occupants’ health symptoms with their indoor living conditions. An IoT architecture for IAQ supervision which incorporates cost-effective air quality, temperature and humidity sensors is proposed by [81]. This system integrates a Raspberry Pi 2 microcontroller and the data collected are saved in a cloud platform. The proposed system provides real-time air quality index data, incorporates e-mail notification features, as well as web compatibility for data consulting. The authors of [82] present an end-to-end IAQ supervision system which provides CO_2_, CO, sulfur dioxide (SO_2_), NO_2_, O_3_, chlorine (Cl_2_), temperature, and humidity monitoring. The monitored data are stored in the Emoncms open-source IoT platform for real-time supervision and long-term storage. This solution uses a hybrid IoT/WSN architecture, the gateway is based on Raspberry Pi, and the sensor nodes are based on the Waspmote microcontroller.

Using real-time air quality monitoring systems, it is possible to identify unhealthy outdoor and indoor scenarios which can be assertively intervened. The proposed solution provides a useful tool for air quality management for ELE of smart cities. CO_2_ assessment leads to several benefits for public health and productivity as the city, or building manager can improve the air quality by planning interventions to decrease the pollution load [83].

### Exposure to Air Pollution

Exposure can be defined as the contact between an airborne pollutant and a surface of the human body. Therefore, this phenomenon requires two events at the same time: an airborne contaminant considering a particular location and time, and the presence of an individual at that location and time [84]. Air pollution exposure also affects beneficial and healthy practices, such as bicycling in urban areas [85]. A study conducted in central city neighbourhoods on the Island of Montreal concluded that cyclists’ exposure levels to air pollution are significant and can be associated with health and safety risks [86].

Air pollution mapping and measurement have several challenges [87]. On the one hand, several pollutants affect air quality at different levels. On the other hand, there are several limitations regarding the insufficient funding and political questions in the design of aggressive policies for air pollutant emission reduction [88]. Mapping real-time air quality using mobile computing technologies promote higher spatial coverage when compared to fixed air quality stations. The fixed stations have relevant limitations since the monitoring data do not provide a continuous time-series of measurements [88]. Moreover, the fixed air quality stations typically have relatively long measurement times, such as an hour or even more [89]. These fixed stations present another significant limitation since they are typically installed in background locations at a relative distance from busy roads and can provide a distorted impression of air pollution exposure [89].

The use of mobile computing technologies, in general, and smartphones, in particular, to handle and process air pollution exposure data lead to several advantages. On the one hand, smartphones are widely used by most people. On the other hand, they include several sensors and communication protocols for data transmission. Furthermore, the mobile devices have processing capacities which enable the visualization and analysis of air pollution measurement data [90].

## 3. Materials and Methods

Air quality is a fundamental challenge for public health and well-being as it is accountable for several health symptoms [91]. Air pollution depends on the concentration of numerous chemical components such as, radon decay products, CO, CH_2_O, NO_2_, and aeroallergens which are correlated to health complications [92]. Therefore, air quality must be measured in real-time to provide a spatial-temporal dataset for enhanced data analytics and visualization.

On the one hand, temperature and humidity monitoring systems are already integrated into our daily routine as they are incorporated both in outdoor spaces and in the cabin of vehicles as people are more sensitive to these types of parameters. Poor thermal comfort conditions are easily detected by people [93]. On the other hand, poor air quality scenarios are only detected by people under extreme conditions. Typically, poor air quality is detected by short-term exposure effects such as eyes, nose, and throat irritation, coughing, chest tightness and shortness of breath [94].

It is essential to adopt effective systems to monitor air quality to alert the user in real time and provide effective behavioural changes for ELE. This is particularly relevant for several specific groups, such as people with cardiovascular disease, coronary artery disease or heart failure, specific lung diseases such as asthma or chronic obstructive pulmonary disease problems, pregnant women, older adults and people under the age of 14 years [95,96,97,98].

**Figure 1:**
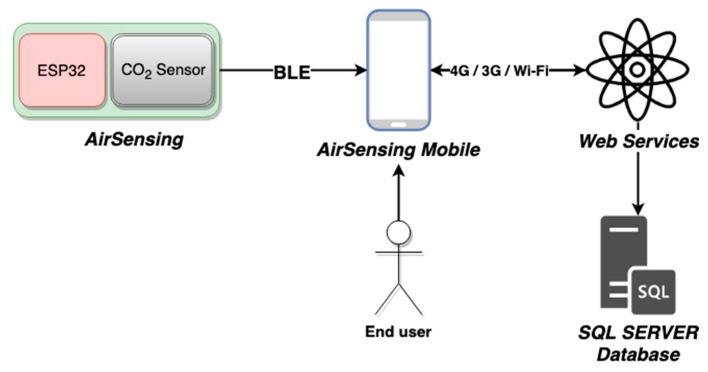
System architecture.

Taking into account this critical public health challenge, the authors propose a portable air quality monitoring system which can measure and map the CO_2_ concentration in real-time and incorporate mobile computing technologies for data analytics and visualization considering the geographical coordinates. The hardware prototype does the data collection and they are sent by BLE to the mobile phone, which is responsible for the communication of the data to a backend.NET application for data storage using an MS SQL Server database. For data consulting, a mobile application was created using the Swift programming language (Figure 1).

**Figure 2:**
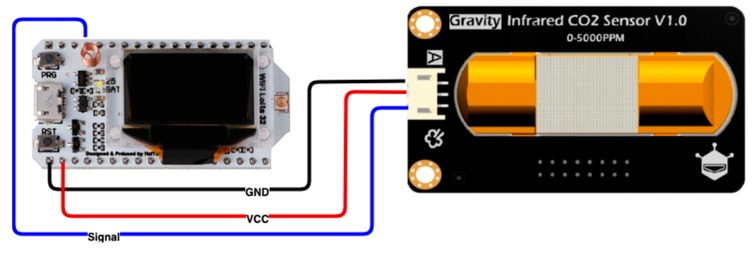
Cyber-physical system.

This solution provides a dataset of CO_2_ concentration in real-time which can aid the city or building manager in providing an efficient air quality assessment regarding the detailed spatial-temporal information of indoor or outdoor spaces, as well as in the projection of strategies and policies to increase people’s health and well-being. The system is distributed into three segments: the cyber-physical system, which is responsible for data collection, a mobile computing system which supports the data upload, and a backend application which is responsible for data storage. The cyber-physical system prototype is represented in Figure 2.

This cyber-physical system is based on the ESP32 microcontroller and uses a Heltec Lora 32; an IoT dev-board developed by Heltec Automation, which integrates an ESP32 and supports Wi-Fi, BLE and LoRa communication technologies, a Li-Po battery management system and 0.96″ OLED. The ESP32 is a 240 MHz Tensilica LX6 dual-core with 520 KB SRAM, and the LoRa chip is an SX1276. This microcontroller incorporates 3 UART; 2 SPI and I2C inputs, a 12 bits ADC, an 8-bits DAC and 29 general GPIO for sensor and actuators connections. The display is a 0.96 inch 128 × 64 OLED, which incorporates an 8 MB SPI FLASH, a micro USB for data programming and the microcontroller dimensions are 50.2 × 25.5 × 9.74 mm.

The sensing unit is designed using the DFRobot CO_2_ analogue infrared sensor. This sensor can measure CO_2_ data from 0 to 5000 ppm. It is based on non-dispersive infrared technology and has a five-year service life. Moreover, this sensor integrates temperature compensation and support analogue output. It has several relevant specifications, such as high stability, low power consumption, fast response, high sensitivity and long life. Table 1 presents the complete sensor specification.

The selection of the MH-Z14 CO_2_ sensor module was conducted considering not only the cost of the system but also the accuracy of the monitored data. This sensor accuracy is enough considering the thresholds defined for the CO_2_ levels by the competent authorities. The proposed solution can be improved by adding other sensors to monitor specific chemical compounds and air pollutants. Moreover, it is relevant to mention that the main objective of the research was to test the proposed IoT approach and system architecture. The hardware cost is an estimated € 85.76 (Table 2).

On the one hand, CO_2_ levels are relevant for indoor and outdoor air quality assessment. High levels of CO_2_ inside classrooms have been studied for several years [99,100,101,102,103]. On the other hand, outdoor air quality depends on the concentration of several substances such as particles, NO_2_, hydrocarbons, CO and O_3_, which derive from combustion sources [104]. Outdoor air quality not only depends on the pollutant emissions, but it also affected by the current ventilation and meteorological conditions [105]. Low wind and low convention promote the accumulation of pollutants. In these scenarios, the levels of CO_2_ also increase [106]. Therefore, a direct relation between CO_2_ and outdoor pollution produced by traffic and industry is also verified [107].

Air quality assessment can be done using multiple sensors to monitor each air pollutant [108]. However, the design of a cost-effective system that incorporates numerous sensors is not possible since the addition of sensors will increase the overall cost of the system. The use of various sensors also increases maintenance procedures [109]. PM sensors are based on optical sensors which need to be regularly cleaned to provide accurate readings [110]. Nitrogen sensors need to be frequently calibrated and replaced [111]. Furthermore, cost-effective sensors for the dedicated monitoring of each chemical composition presented in the air do not exist.

Nowadays, CO_2_ sensors are reliable, accurate and maintenance-free [112]. CO_2_ sensors can be used to provide an efficient and practical association between combustion-related emissions and outdoor air quality conditions. The correlation between outdoor air quality and the particular concentration of air pollutants depends on their source as diesel cars produce higher emissions of nitrogen dioxide than gasoline vehicles, for example [113]. This relation also depends on the season and the urban area [114]. Nevertheless, high levels of CO_2_ are always a sign of high pollutant emissions considering the air exchange, which is therefore, an indication of the high risk for pollutants. Likewise, a low CO_2_ level indicates that air pollutants are not accumulated and reveals good air quality scenarios [115].

**Figure 3:**
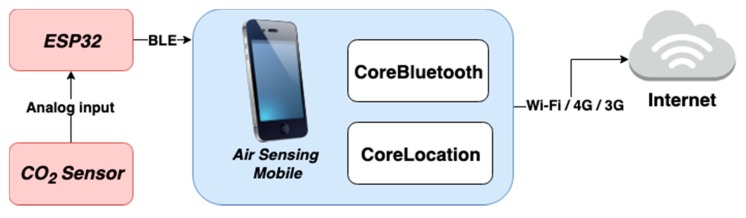
System communication architecture.

In particular scenarios and applications, there is no significant need for high accuracy solutions because a qualitative assessment is sufficient to promote health and well-being. However, the proposed system incorporates a reliable CO_2_ sensor to address the applications where accuracy is relevant. More important than the accuracy properties is to provide a portable solution for IAQ monitoring, which can map and measure the CO_2_ levels in both indoor and outdoor environments. The cyber-physical system firmware was developed using the Arduino Core which is an open-source framework which offers Arduino functions and libraries support for the ESP32 microcontroller. This hardware is responsible for data acquisition and uses built-in BLE technology to communicate with a smartphone. The smartphone is responsible for GPS data handling. Moreover, the mobile application uses web services for data transmission and storage on an MS SQL Server. The proposed method records the CO_2_ levels every 120 seconds (Figure 3). For testing proposes, a smartphone application was created using Swift programming language and Xcode Integrated Development Environment. This mobile application supports iOS 12 and above. This app, named *AirSensingMobile,* offers real-time data access and turns possible to check the location where this data is recorded.

BLE is a wireless communication technology developed for battery-powered systems [116]. BLE uses the 2.4 GHz radio frequency band and consumes less power than ZigBee [117,118]. BLE technology is the most used communication technology for the connection of hardware accessories with mobile phones [119]. Consequently, BLE communication technology is a suitable approach for IoT applications and was selected to provide data communication between the cyber-physical system and the mobile application. The Core Bluetooth is Apple’s framework to send and receive messages using BLE technology on iOS applications. GPS is a space-based satellite navigation system which provides location and time information anywhere on Earth. This positioning system is used for several applications in military, civil, and commercial fields. Today, the majority of smartphones support GPS features, on iOS operating systems. CoreLocation is the framework responsible for GPS data acquisition.

Most of the air quality monitoring systems for indoor and outdoor environments available in the market are expensive and do not support spatial-temporal properties. Some of these solutions provide only data consulting on the equipment’s display or provide data download procedures for further analysis. However, portability and real-time data sharing are relevant to provide healthy environments for the occupants. Furthermore, the location of the data collected is crucial in specific fields, such as the vehicles’ cabins monitoring. Thus, the proposed method presents a portable air quality monitoring system with integrated wireless communication technology which provides an effective method to map and measure the air quality levels for further analysis to plan interventions for ELE.

## 4. Results and Discussion

The proposed solution was developed to provide air quality sensing in both indoor and outdoor environments. The mobile application offers data consulting in terms of geographic location. For testing purposes, the cyber-physical system was powered using a 5V 10,000 mA power bank. Real-time air quality supervision must be considered as a relevant tool to support decision-making not only to plan behavioural changes inside buildings but also to ensure healthy outdoor conditions through the correct planning and flow of traffic. Usually, the outdoor air quality levels are not measured in real-time using mobile air pollution monitoring technologies. The outdoor air quality is typically monitored by stationary monitoring units with a fixed location. These monitoring units have a high cost of installation and operation [88]. However, real-time monitoring procedures applied outdoor can help the identification of specific locations where the air quality conditions are defective to improve public health and safety. Nowadays, mobile phones usage time in the western world has increased considerably. Smartphones are used for numerous daily routine activities and support several communication technologies but also significant processing power and data visualization tools. The majority of smartphones incorporate GPS [120]. In total, 70% of the Netherlands population and 90% of adolescents have smartphones [121]; 40% of the German population use a smartphone [122], and in the United Kingdom, 51% of adults also have smartphones [123].

**Figure 4:**
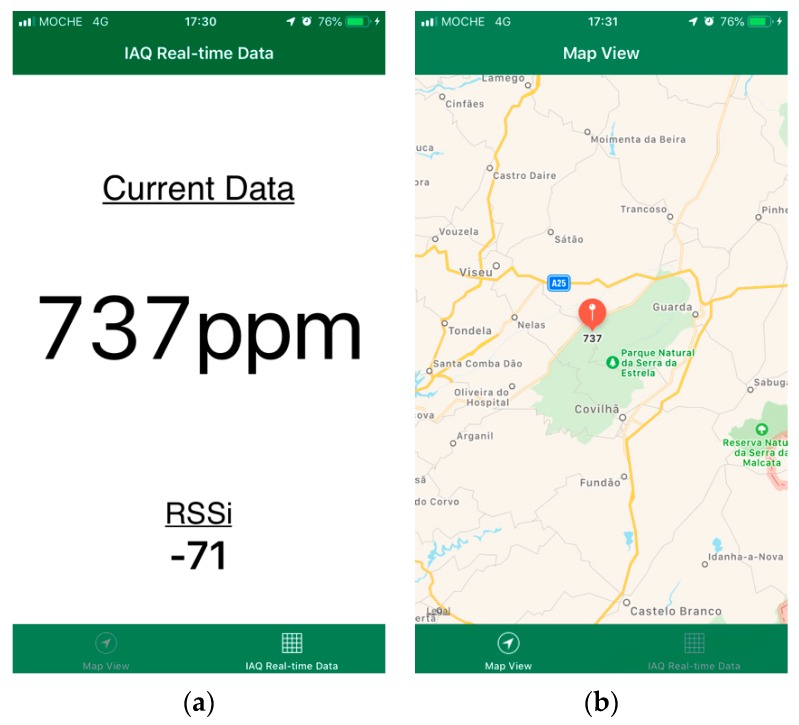
(**a**) CO_2_ collected data; (**b**) corresponding map view.

Furthermore, mobile computing technologies have offered ubiquitous Internet connection which leads to numerous daily routine changes. Therefore, a mobile application was proposed to provide an effective method for the user to carry their air quality sensing data for continuous use. Figure 4a presents the last data collected by the system. Thus, the user can consult the CO_2_ data collected but also check the BLE RSSI from the connection between the cyber-physical system and the smartphone. Figure 4b shows the corresponding location from the data collected. Using this method, it is possible to measure and map the air quality sensing in real-time for ELE in smart cities.

The proposed method supports numerous advantages regarding modularity, size, portability, cost-effective and easy installation. The mobile application provides an easy and accessible method for data analysis and visualization. On the one hand, the proposed architecture provides a relevant dataset for environmental management. Using the proposed system, it is possible to detect defective air quality scenarios and plan interventions for ELE. Therefore, this mobile computing solution can be assumed as a relevant decision-making tool to define strategies to increase air quality conditions and to guarantee the efficiency of these methods.

**Figure 5:**
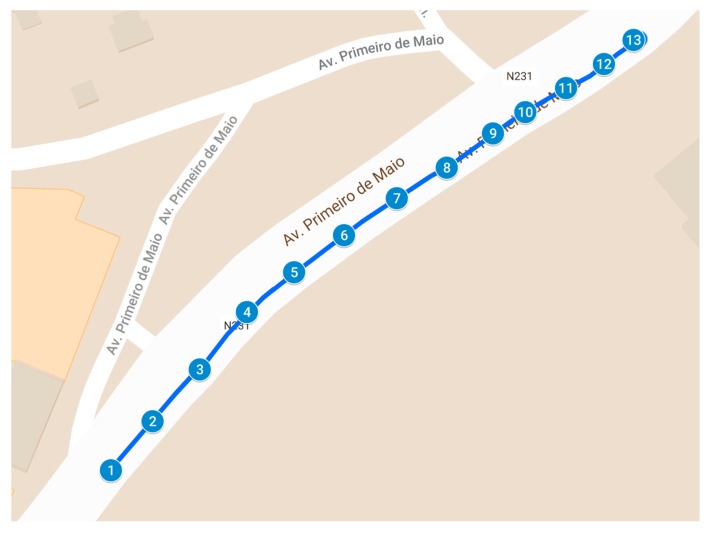
Mapping of CO_2_ concentrations during the tests performed.

Figure 5 presents the mapping of the monitored locations in the tests conducted. The numbers of Figure 5 represented the exact location where the CO_2_ concentrations were measured. The details regarding the latitude, longitude, CO_2_ level and time of the data collection are presented in Table 3.

On the other hand, real-time data assessment allows us to conclude that typically the air quality is under demanded standards. The data collected during the tests conducted states that under certain conditions and during some specific times of the day, air quality levels can be very different from healthy standards. Real-time air quality monitoring methods combined with the use of mobile computing technologies for data consulting, increase the population’s attention to the critical air quality problem and supports them to preserve healthier environmental conditions.

Air quality sensing is a relevant requirement for enhanced public health and well-being. Consequently, the design and development of new cost-effective methods for air quality monitoring based on open-source and mobile computing technologies are a trending research theme. Table 4 presents a summarized comparison review of the air quality supervision solutions described above.

Regarding the systems presented in Table 4, the proposed method incorporates several advantages. Compared to the architecture proposed by the authors of [82], which is based on wireless sensor networks (WSN), the proposed method eliminates the necessity to configure the sensor nodes and gateways. Moreover, the proposed system supports easy installation as it is only necessary to configure the BLE communication between the cyber-physical system and the mobile device. Furthermore, the proposed method provides air quality measurement and mapping. The geographical location of the monitored data leads to a significant advantage as it is possible to track the air quality evolution on the move. This system can be installed outside vehicles to monitor air quality while travelling. This functionality is not incorporated in any of the methods described in Table 4 as none of the systems support portability. The proposed system is based on the ESP32 microcontroller. First, the ESP32 supports Wi-Fi, BLE and LoRa communication technologies. Second, this microcontroller supports 240 MHz of clock speed which contrasting with the 16 MHz of the Arduino [79] and the 80 MHz of the ESP8266 [63,64,80].

The air quality supervision conducted in most places is based on random sampling using high-cost professional equipment. Nevertheless, the information collected by those kinds of systems is limited as they are devoid of spatiotemporal behaviour. The air quality monitoring systems presented by [124,125,126,127,128] are portable and incorporate information storage on the device itself but do not support real-time data consulting features or mobile computing compatibility. These portable solutions have high-cost and require specific procedures using proprietary software to extract the data collected for further analysis and visualization. Table 5 presents a summary of the portable solutions available in the market.

The solutions presented in Table 5 provide portability features; however, the data collected cannot be consulted in real-time to be consulted by the authorities to support decision making on possible interventions for enhanced public health. These solutions present accurate relative readings and efficient sensor range and resolution. The maximum sensor error value depends on the sensor measurement range since the accuracy of the sensor is directly influenced by the sensor reading value. Considering a CO_2_ concentration of 1000 ppm, the maximum error value is 50 ppm VOLTCRAFT CM 100 [126]; is 80 ppm for the DZSF AR8200 [124], Reeseiy CO2 [125] and ROTRONIC CP11 [127]; and is 100 ppm for the Extech CO230 [128]. In the worst-case scenario, the maximum error value is 200 ppm for the VOLTCRAFT CM 100 [126]; is 280 ppm for the ROTRONIC CP11 [127]; 530 ppm for the DZSF AR8200 [124] and Reeseiy CO2 [125]; and is 550 ppm for the Extech CO230 [128]. However, the proposed solutions available on the market require data extraction and classification procedures before can be analyzed. Accordingly, the design of a portable air quality monitoring methods based on state-of-the-art open-source technologies that provide real-time data sharing and spatiotemporal information is essential to promote public health.

Therefore, the method proposed by the authors is a reliable and effective solution for real-time air quality systems which combine portability, scalability and modularity features as essential benefits related to current solutions. The selection of the sensor was conducted, taking into consideration the accuracy and cost of the system. The principal objective of the study was to validate the IoT approach. Consequently, the focus of the research was the design and development of portable real-time data acquisition system for air quality measurement and mapping. The CO_2_ sensor was chosen because this type of gas can be used for both indoor and outdoor air quality assessment. Moreover, the CO_2_ data can support clinical analysis conducted by health professionals. In the future, air quality sensing will be incorporated in every living environment as it allows an accurate validation of healthy living environments for enhanced public health and well-being.

The proposed method can be used to monitor the air quality inside vehicles on the move to create a dataset which will be relevant to identify poor air quality scenarios in particular locations and often to better understand the effect and implications of the outdoor air quality parameters on the vehicle’s occupants. In particular, air quality monitoring is of utmost importance in public transports due to the number of occupants and also the ventilation methods used in this kind of vehicles. The proposed system can be easily installed inside public vehicles to perceive the IAQ, considering not only the location of the vehicle but also the number of occupants with reference to the spatiotemporal information. This data will open a relevant discussion to the quality perceived inside public vehicles at specific hours of the day and promote policies to promote public health.

Based on the results achieved the proposed method can be assumed as an effective method for real-time air quality assessment. Moreover, air quality data can be investigated to detect poor air quality patterns for effective intervention planning in smart cities. Furthermore, these data can be used to correlate the identification of multiple defective situations with the population’s habits and behaviours that reduce air quality. The principal objective of the presented system is the creation of a practical tool kit to guarantee and cost-effectively promote air quality standards. This solution provides a relevant dataset to support interventions and can be used for clinical decision support. Air quality data can be used to support medical reports because it is possible to correlate the health complications of specific groups of people who share the same living environment with the location air quality data.

Nevertheless, the main limitation of the proposed method is associated with the main challenge of the standard GPS systems and relies on the post-processing of the GPS data. In most indoor environments, it is not possible to collect GPS data when the satellite signal is down. However, the use of mobile technologies brings a significant advantage since it is possible to get using the Internet provider when GPS data reception fails. Furthermore, the proposed method has limitations associated with the sensors used. The main limitations of the air pollution sensor employed in this study are related to its preheating and response time.

## 5. Conclusions

IoT, AAL and mHealth research fields contribute to the design and development of enhanced architectures for smart living and public health. Moreover, the relevant capabilities for sensing and connecting provided by mobile computing technologies provide an adequate structural base for real-time monitoring methods. This document has proposed a real-time air quality monitoring system based on IoT, which incorporates a cyber-physical system for data collection and mobile computing software for data consulting. This architecture makes possible the measurement and mapping of air quality levels considering the spatial-temporal information. The proposed method connects different technological fields as IoT, mHealth, AAL and mobile computing. Further, this solution provides a low-cost and efficient method for air quality monitoring and integrates a smartphone application for data access and enhanced data analysis. The main contribution of this paper is the proposal of a low-cost system which can be easily installed and configurated inside vehicles to monitor IAQ on the move. The proposed methods incorporate numerous benefits when associated with other supervision solutions such as modularity, portability, easy installation and configuration, as well as mobile computing technologies integration. The collected data can support the design of strategies and policies to increase air quality. The tests conducted confirm the implementation of the proposed architecture and present a relevant contribution to the air quality supervision systems available in the literature. However, the proposed method has limitations and needs additional experimental validation to increase sensor calibration and accuracy.

In the future, numerous improvements are planned to adjust this method to particular use cases such as the installation of the system on the exterior of public vehicles which are always moving around the city and can measure and map air quality in real-time. In this context, it will be possible to monitor the city air quality continually considering the geographical coordinates to create a relevant dataset which can be used to regulate traffic at specific hours of the day and promote public health. It is also planned to perform additional tests to evaluate the accuracy of the sensors with different types of technologies. Air quality monitoring allows the detection of unhealthy behaviours in real-time and should be incorporated in all living environments and be a fundamental part of the daily routine to promote health and well-being. The authors believe that architectures like the one proposed will provide ELE in smart cities.

## Figures and Tables

**Table 1:** MH-Z14 sensor specification.

Specification	Value
Operating Voltage	4.5 ~ 5.5V DC
Average Current	<60mA at 5V
Peak Current	150mA at 5V
Output Signal	0.4–2 V
Measuring Range	0~5000 ppm
Accuracy	± (50ppm 3% reading)
Preheating Time	3 min
Response Time	120s
Working Temperature	0 ~ 50 ℃
Working Humidity	0 ~ 95%
Sensor lifespan	>5 years
Size	37 mm × 69 mm

**Table 2:** Cost of the system.

Component	Cost
ESP32	24.15 €
MH-Z14	52.11 €
Cables and box	9.50 €
Total	85.76 €

**Table 3:** Measurement of CO_2_ concentrations during the tests performed.

Marker	Latitude	Longitude	CO_2_ (ppm)	Date and Time
1	40.41641	−7.70737	511	14 December 2019 17:02
2	40.41651	−7.70725	481	14 December 2019 17:04
3	40.41663	−7.70712	484	14 December 2019 17:06
4	40.41663	−7.70712	439	14 December 2019 17:08
5	40.41684	−7.70684	460	14 December 2019 17:10
6	40.41692	−7.7067	424	14 December 2019 17:12
7	40.41701	−7.70654	510	14 December 2019 17:14
8	40.41707	−7.7064	501	14 December 2019 17:16
9	40.41715	−7.70627	511	14 December 2019 17:18
10	40.4172	−7.70617	670	14 December 2019 17:20
11	40.41725	−7.70605	716	14 December 2019 17:22
12	40.4173	−7.70594	531	14 December 2019 17:24
13	40.41736	−7.70586	453	14 December 2019 17:26

**Table 4:** Summarised comparison review of air quality monitoring solutions.

MCU	Sensors Unit	Architecture	Low Cost	Open-Source	Connectivity	Data Consulting	GPS	Portability
ESP8266 [64]	CO_2_	IoT	√	√	Wi-Fi	Web/Mobile	×	×
ESP8266 [63]	NH_3_, CO, NO_2_ C_3_H_8_, C_4_H_10_, CH_4_, H_2_ and C_2_H_5_OH	IoT	√	√	Wi-Fi	Mobile	×	×
Arduino UNO [79]	CO_2_, PM, light, temperature and relative humidity	IoT	√	√	Wi-Fi/BLE	Smartwatch	×	×
ESP8266 [80]	PM, CH_2_O, temperature and relative humidity	IoT	√	√	Wi-Fi	Web/Mobile	×	×
Raspberry Pi 2 [81]	air quality index, temperature, relative humidity	IoT	√	√	Wi-Fi	Web	×	×
Waspmote (sensor node) Raspberry Pi 2 (coordinator) [82]	CO_2_, CO, SO_2_, NO_2_, O_3_, Cl_2_, temperature, and relative humidity	WSN/IoT	√	√	Wi-Fi	Web	×	×
Proposed method	CO_2_	IoT	√	√	BLE	Mobile	√	√

MCU: microcontroller; √: support; ×: does not support.

**Table 5:** Portable CO_2_ monitoring systems available on the market.

Solution name	Range (ppm)	Resolution (ppm)	Error (ppm)	Price (EUR)
DZSF AR8200 [124]	350–9999	5	± (30 + 5% reading)	377.38
Reeseiy CO2 [125]	0–9999	1	± (30 + 5% reading)	111.42
VOLTCRAFT CM 100 [126]	0–4000	1	±5% of reading	302.78
ROTRONIC CP11 [127]	0–5000	1	± (30 + 5% reading)	373.39
Extech CO230 [128]	0–9999	1	± (50 + 5% reading)	239.00
